# Oncogenic Potential and Clinical Implications of Giant Endometrial Polyps: A Case Report and Literature Review

**DOI:** 10.1155/2018/8753463

**Published:** 2018-06-07

**Authors:** Brittany van Staalduinen, Andrew Stahler, Catherine Abied, Rita Shats, Nisha A. Lakhi

**Affiliations:** ^1^Department of Obstetrics and Gynecology, Richmond University Medical Center, 355 Bard Avenue, Staten Island, NY 10310, USA; ^2^Department of Obstetrics and Gynecology, New York Medical College, Valhalla, New York, USA

## Abstract

Endometrial polyps exceeding 4 centimeters in length are exceedingly rare and are termed “giant polyps.” We describe two patients that presented to our hospital with giant endometrial polyps. Clinical implications and oncologic potential of giant endometrial polyps are discussed. Risk factors of oncologic transformation include advanced age, menopausal status, obesity, diabetes, arterial hypertension, use of tamoxifen, and size greater than 1.0 centimeter. A literature review of all documented cases of giant endometrial polyps is presented and management strategies for counseling and polypectomy are reviewed.

## 1. Introduction

Endometrial polyps are localized overgrowths of the endometrial lining of the uterus. Microscopically, they are composed of varying amounts of glandular tissue, stroma, and blood vessels covered by an epithelium. Their size can range from a few millimeters to beyond 5 centimeters in length. Endometrial polyps larger than 1 cm are termed “large polyps,” and those that are greater than 4 cm, which are exceedingly rare, are called “giant polyps” [1].

The exact incidence of endometrial polyps is unknown; however, in women with dysfunctional uterine bleeding, the prevalence of endometrial polyps ranges from 10% to 24% [2]. 10% to 25% of symptomatic polyps may contain hyperplastic foci and malignant transformation has been observed in about 0 to 12.9% [2]. Although evidence in the literature defining factors linked to malignant transformation is contradictory, advanced age, menopausal status, obesity, diabetes, arterial hypertension, and use of tamoxifen have reached statistical significance in varying reports [3–5]. Additionally, polyp size has been found to be a predictive factor. B. P. Lasmar and R. B. Lasmar [6] found that endometrial polyps larger than 1.5 cm were associated with hyperplasia, while a separate report by Wang et al. [3] identified that polyps measuring more than 1.0 cm were associated with malignancy.

We describe two patients that presented to our hospital with giant endometrial polyps and present a literature review of other similar cases. The oncogenic potential of large polyps is evaluated and evidence-based management strategies are discussed.

## 2. Case Series

### 2.1. Case #1

FM is a 70-year-old postmenopausal black female who presented with a chief complaint of intermittent vaginal spotting since the age of 50. She had not sought gynecologic care for several years. FM reported two episodes of vaginal spotting over the previous year, each lasting approximately one week in duration. Otherwise, she was asymptomatic and denied any recent intercourse or trauma to the vaginal area. Her past medical history was significant for poorly controlled hypertension, poorly controlled type 2 diabetes mellitus, osteoarthritis, angina, vitamin D deficiency, and glaucoma. She reported a long history of normal Pap smears over the course of her life and had one full-term normal spontaneous vaginal delivery. Family history was significant for breast cancer in her mother at the age of 60 years. She denied any family history of uterine cancer, colon cancer, or ovarian cancer.

**Figure 1:**
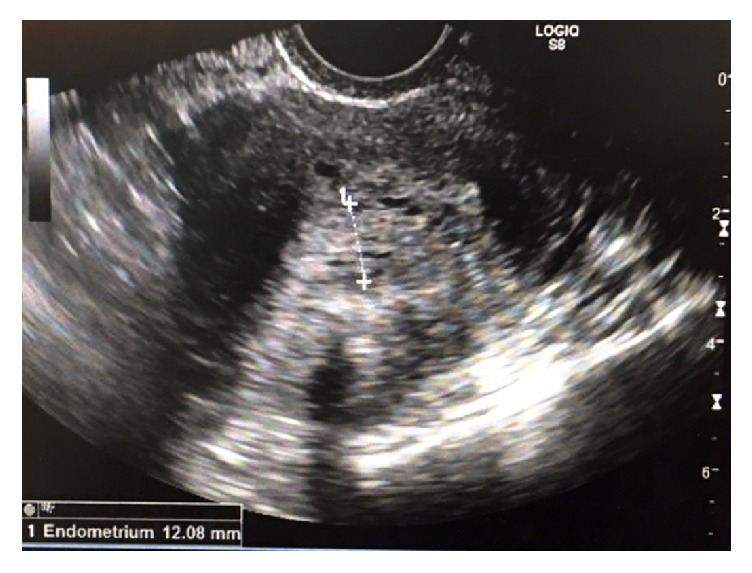
Ultrasound imaging of uterus demonstrating a 12.1 mm endometrial stripe.

**Figure 2:**
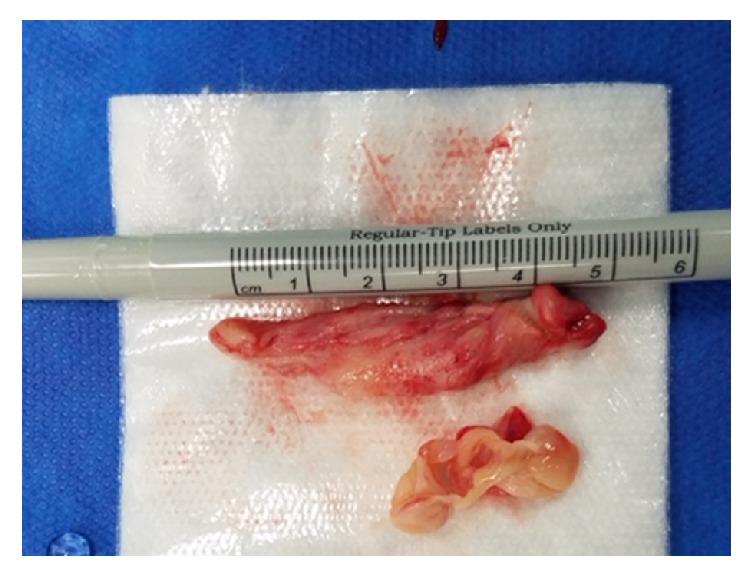
Gross specimen of polyp from case #1.

**Figure 3:**
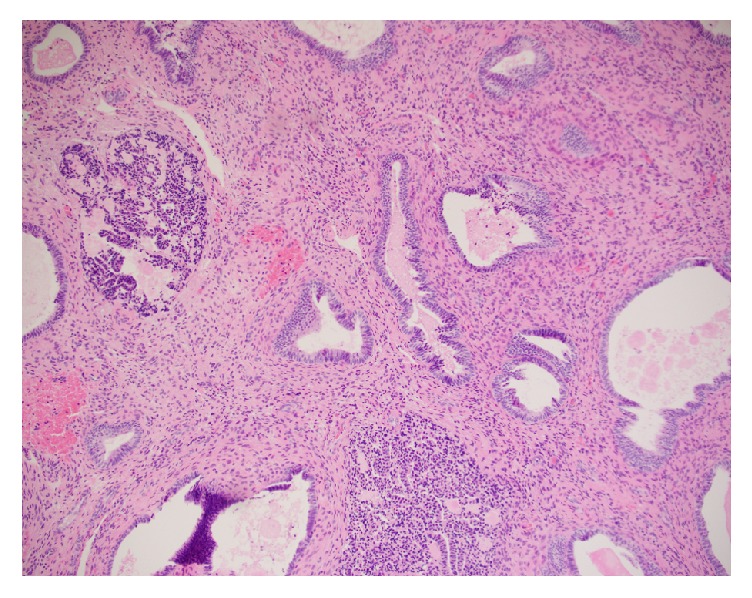
Microscopic section of polyp from case #1. H&E stain, demonstrating inactive endometrium, few glands, fibrotic stroma, and dilated, thick-walled blood vessels.

On initial evaluation, FM was alert and oriented and well nourished, with a body mass index (BMI) of 28 kg/m^2^. On physical examination, her vaginal mucosa was atrophic. A 1 cm polyp that originated from the endometrial cavity was found to be protruding from the cervical os and was friable to the touch. On bimanual exam, the uterus was anteverted, smooth, and mobile without palpable adnexal masses. Transvaginal ultrasound confirmed an anteverted uterus measuring 9.1×5.2×7.1 cm with a thickened, heterogeneous endometrial echo measuring 12.1 mm with multiple cystic small spaces (Figure 1). A vascular signal was seen throughout the thickened endometrium and a partially calcified leiomyoma in the left uterine body was also noted. An endometrial biopsy was obtained and showed rare superficial fragments of inactive endometrial tissue. Due to postmenopausal bleeding, the patient was booked for operative hysteroscopy, dilation and curettage, and polypectomy. On hysteroscopy, a large polyp was seen arising from the anterior uterine wall. Using operative hysteroscopy and hysteroscopic scissors, this polyp, measuring 5.3 cm in greatest dimension, was removed in its entirety (Figure 2). Endometrial curettage was performed after complete removal of the polyp. Final pathology showed inactive endometrium without evidence of hyperplasia (Figure 3). At the two-week postoperative visit, FM reported complete resolution of her symptoms.

### 2.2. Case #2

**Figure 4:**
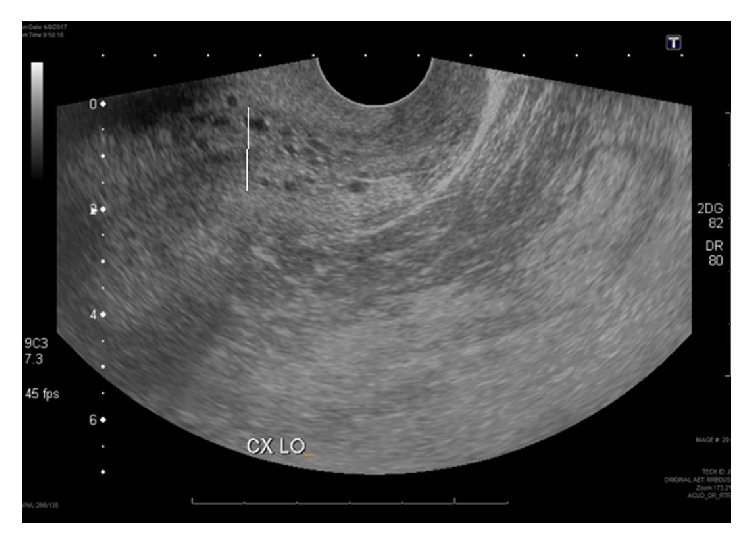
Ultrasound imaging of uterus demonstrating endometrial lining measuring 20 mm containing small cystic appearing areas.

**Figure 5:**
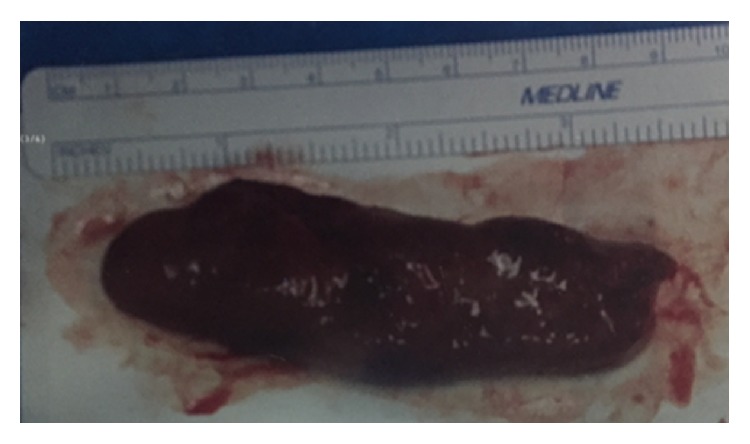
Gross specimen of polyp from case #2.

**Figure 6:**
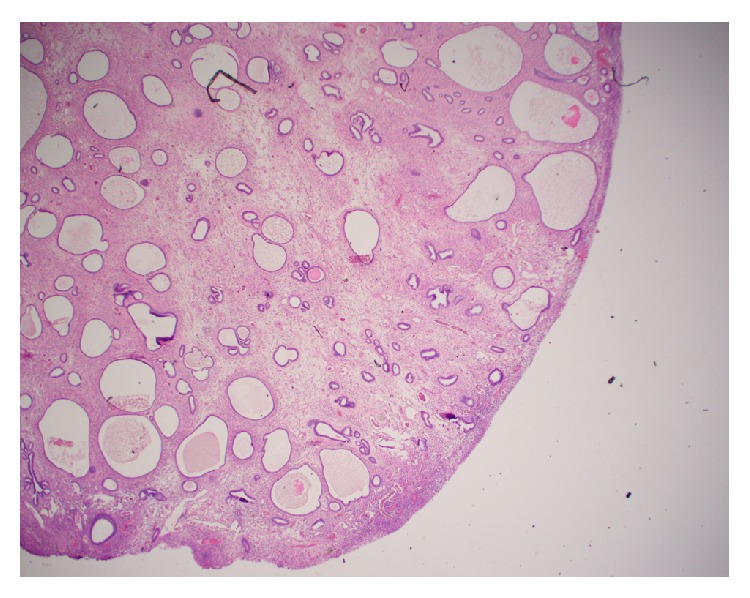
Microscopic section of polyp from case #2. H&E stain, low-power photomicrographs showing intact polypoid tissue cystic dilated glands without evidence of hyperplasia, fibrotic stroma, and dilated thick-walled vessels.

HA is a 66-year-old obese postmenopausal Hispanic female who presented with a chief complaint of intermittent vaginal spotting of a duration of eight months. Past medical history was significant for poorly controlled hypertension, aortic stenosis, hypercholesterolemia, and glaucoma. She reported a history of normal Pap smears throughout her life, the last one being one month prior to presentation. She had two full-term vaginal deliveries and one cesarean delivery. HA denied any significant family history of malignancy. On initial examination, she was noted to be alert and oriented and was obese with a BMI of 39.5 kg/m^2^. The external genitalia appeared normal and without lesions. A polyp was seen protruding 2 cm from the cervical os. On bimanual exam, the uterus was noted to be mobile, anteverted, and smooth, with no palpable adnexal masses. Transvaginal ultrasound revealed a 9.6 × 5.9 × 5.7 cm uterus with endometrial lining measuring 20 mm with small cystic appearing areas (Figure 4). Due to the large polyp protruding from the cervical os, the patient was booked for operative hysteroscopy, dilation and curettage, and polypectomy. The polyp was grasped with forceps and removed from its base prior to the hysteroscope being introduced to the uterine cavity. The polyp measured 8 cm in its greatest dimension. Hysteroscopy revealed areas of polypoid tissue which were removed under direct visualization (Figure 5). Endometrial curettage was then performed. Final pathology revealed inactive endometrium with no hyperplasia or atypia (Figure 6). At her two-week postoperative visit, she reported a resolution of her symptoms.

## 3. Discussion

Endometrial polyps are found in both premenopausal and postmenopausal women. Patients may either be asymptomatic at the time of diagnosis or present with abnormal bleeding patterns, including intermenstrual bleeding, menorrhagia, or postmenopausal bleeding [1]. The exact cause of endometrial polyps is unknown, but estrogenic activity appears to play a crucial role in their pathogenesis and growth [2]. Several molecular mechanisms have been proposed to play a role in the development of endometrial polyps. These include overexpression of endometrial aromatase, unbalanced activity between estrogen and progestin, inhibition of apoptosis, certain gene mutations that favor endometrial proliferation, and cellular mechanisms linked with inflammation [3, 4].

Previous case series indicate that malignancy occurs within 0% to 12.9% of endometrial polyps [4]. Although there is no consensus in the literature on the exact risk factors that are associated with malignant transformation of polyps, most authors agree that the risk of malignancy is increased with age and menopausal status and with the presence of symptomatic bleeding [1, 2]. Larger endometrial polyps also have been shown to be a risk factor for premalignant or malignant pathology, with authors advocating a cut-off point of 1.0 to 1.8 cm diameter as a risk factor [3–6].

Wang et al. retrospectively reviewed consecutive cases of patients that underwent hysteroscopic removal of an endometrial polyp and correlated malignant and premalignant lesions to clinical risk factors [3]. Of the 766 patients, polyps were histologically benign (no atypia) in 96.21% of patients, hyperplasia with atypia was identified in 3.26% of cases, and invasive endometrial carcinoma was present in only 0.52% of patients. Independent variables that were significantly related to premalignant and malignant polyps included polyp diameter greater than 1.0 cm, menopausal status, and abnormal uterine bleeding. In this report, hypertension, diabetes mellitus, body mass index, and use of tamoxifen were not found to be associated with the malignant transformation of polyps [3].

Ferrazzi et al. compared the prevalence of hyperplasia and malignancy in endometrial polyps among a cohort of 1,552 asymptomatic postmenopausal patients in comparison to a similar cohort of 770 postmenopausal patients that presented with abnormal uterine bleeding [15]. The prevalence of atypical hyperplastic polyps was 1.2% in asymptomatic versus 2.2% in symptomatic patients (*P* < .005). One single case of stage 1 grade 1 endometrial carcinoma was recorded within a polyp with a mean diameter 4 cm in an asymptomatic patient. After multivariate analysis, diameter of the polyps was the only variable significantly associated with an abnormal histology (malignancy and atypical hyperplasia) in asymptomatic women with an odds ratio of 6.9 (confidence interval: 2.2–21.4) for polyps with mean diameter > 1.8 cm [15].

Similarly, another retrospective review of 1,136 asymptomatic women that underwent hysteroscopic resection of an endometrial polyp found that polyps with diameters greater than 1.5 cm had hyperplasia rates of 14.8% compared with 7.7% in the group with smaller polyps (*P* < 0.5) [6]. Comparable to the above findings, Ben-Aire et al. assessed 430 women with endometrial polyps undergoing hysteroscopic resection and found hyperplasia without atypia in 11.4% of cases, hyperplasia with atypia in 3.3% of cases, and malignant conditions in 3.0% of cases [5]. Older age, menopause status, and polyps larger than 1.5 cm were associated with significant premalignant or malignant changes [8]. Interestingly, the presence of postmenopausal or irregular vaginal bleeding was not a predictor of malignancy in this study [8].

Giant endometrial polyps larger than 4 cm in diameter are exceedingly rare.  Table 1 summarizes the management and outcomes of reported cases of giant endometrial polyps in the literature. The mean age of patients with giant endometrial polyps was 66.6 years (range: 55–70). All of the reported patients were postmenopausal. The most common presenting symptom was vaginal bleeding. Of the reported cases, 58% (7/12) were in association with use of a phytoestrogen or selective estrogen receptor modulators such as tamoxifen or raloxifene. In the report by Unal et al., the authors state that the patient consumed a large amount of thyme, a known phytoestrogen [13]. The six highest estrogen binding herbs are soy, licorice, red clover, thyme, turmeric hops, and* Verbena* [13].

Although not on exogenous drugs, the patient described by Narin et al. had several risk factors similar to our patients, including obesity, older age, and hypertension [10]. The patient described by Meena et al. did not present with postmenopausal bleeding and did not use hormones. However, she had several risk factors including postmenopausal status, obesity, diabetes mellitus, and hypertension. Similarly, the patient in the case by Unal et al. did not report vaginal bleeding; her polyp was discovered by an incidental CT scan for complaints of lower back pain. Demographic details were not provided in the report by Çil et al.; however, the patient was of older age [1]. In addition to their menopausal status, both of our patients have medical comorbidities that may be risk factors for malignant transformation of polyps including hypertension, obesity, and type 2 diabetes mellitus. It is interesting to note that 8 of the 12 patients described were of Turkish or Mediterranean origin. It is possible that there are genetic, dietary, or ethnic factors related to the development of large polyps; however, with such a small incidence and limited reporting, no conclusions can be inferred.

The rationale for removing polyps is to exclude malignancy and to relieve symptomatic vaginal bleeding. Management of small asymptomatic polyps may be conservative with follow-up. However, conservative management should be undertaken with caution in postmenopausal patients, patients with any risk factors, or those with polyps measuring greater than 1.0–1.5 cm in size, as there is increased risk for atypical hyperplasia or malignancy [7, 15]. Risk factors for malignancy differ among reports and populations; however, larger size, advanced age, menopausal status, obesity, diabetes, arterial hypertension, and tamoxifen use have been associated with malignancy.

Hysteroscopic polypectomy remains the mainstay of evaluation and operative management of endometrial polyps as the associated morbidity is minimal when compared to a hysterectomy. Operative hysteroscopy allows for visualization of the entire uterine cavity. There are a variety of methods practiced to remove polyps at hysteroscopy (sharp scissors, electrosurgical techniques); however, there are no comparative studies for these methods with regard to efficacy. Therefore, the method of choice should be one that is most familiar to the surgeon. Regardless of which method is employed, removal of the entire polyp, including complete excision of the polyp stalk, should be achieved. Studies have indicated that removal of endometrial polyps by blind curettage is unsuccessful in more than 50% of attempts, and, in many cases, the removal is incomplete [4]. Therefore, blind curettage should not be used as a diagnostic or therapeutic intervention [4]. If malignancy is found within the polyp, the patient should be referred to a gynecological oncology specialist for further staging and management.

It should be emphasized that the clinical implications and oncogenic potential of large and giant endometrial polyps are still unclear in the literature. Information is currently derived from small studies, case series, and case reports. The pathogenesis of endometrial polyps as well as factors leading to oncogenesis is still being elucidated. Therefore, with these limitations in knowledge, caution should be taken when counseling patients that present with large or giant endometrial polyps.

## Figures and Tables

**Table 1:** Summary of reported cases of giant endometrial polyps.

Report	Patient age	Polyp size (cm)	Associated drugs	Management	Pathology
Çil et al. [1] Malatya, Turkey	73	8 × 4 × 3	No	Hysteroscopic polypectomy	No hyperplasia, atypia, or malignancy

Moon et al. Busan, Republic of Korea [7]	58	7.5 × 5.5 × 2.6	Tamoxifen	Total abdominal hysterectomy	No hyperplasia, atypia, or malignancy

Nomikos et al. [8] Piraeus, Greece	74	Diameter 8 cm	Tamoxifen	Total abdominal hysterectomy	Complex hyperplasia with atypia

Kutuk and Goksedef [9] Amasya, Turkey	63	4.5 × 4 × 5.2	Raloxifene	Polypectomy under ultrasound guidance	No hyperplasia, atypia, or malignancy

Narin et al. [10] Adana, Turkey	66	12 × 6 × 5	No	Total abdominal hysterectomy, BSO	No hyperplasia, atypia, or malignancy

Erdemoglu et al. [11] Isparta, Turkey	55	10 × 6 × 3	Tamoxifen	Extraction of mass under general anesthesia	No hyperplasia, atypia, or malignancy

Caschetto et al. [12]	64	9 × 7 × 4.5	Tamoxifen	Total abdominal hysterectomy, BSO	Complex hyperplasia with atypia
Catania, Italy	67	8 × 6 × 3	Tamoxifen	Total abdominal hysterectomy, BSO	Simple hyperplasia, no atypia

Ünal et al. [13] Antalya, Turkey	78	10 × 9	Phytoestrogens	Total abdominal hysterectomy	No hyperplasia, atypia, or malignancy

Meena et al. [14] Delhi, India	65	8.5 × 1.5	No	Hysteroscopic polypectomy	Cystic hyperplasia without atypia

van Staalduinen et al.	70	9.1 × 5.2 × 7.1	No	Hysteroscopic polypectomy	No hyperplasia, atypia, or malignancy
New York, USA (index cases)	66	8 × 6 × 5	No	Polypectomy/hysteroscopy	No hyperplasia, atypia, or malignancy

## Data Availability

The data presented in article is available from the corresponding author upon request.
